# Incidence of immediate postpartum hemorrhages in French maternity units: a prospective observational study (HERA study)

**DOI:** 10.1186/s12884-016-1008-7

**Published:** 2016-08-24

**Authors:** Françoise Vendittelli, Chloé Barasinski, Bruno Pereira, Didier Lémery, the HERA Group, Bernard Branger, Laure Connan, Catherine Crenn-Hébert, Caroline Da Costa Correia, Michel Dreyfus, Paul Lefèvre, Anne Legrand

**Affiliations:** 1 Pôle Femme et Enfant, Centre Hospitalier Universitaire de Clermont-Ferrand, Clermont-Ferrand, 63003 France; 2 Université d’Auvergne, EA 4681, PEPRADE (Périnatalité, grossesse, Environnement, PRAtiques médicales et DEveloppement), Clermont Université, Clermont-Ferrand, France; 3 AUDIPOG (Association des Utilisateurs de Dossiers Informatisés en Pédiatrie, Obstétrique et Gynécologie), Faculté de médecine RTH Laennec, Lyon, France; 4 Direction de la Recherche clinique, Centre Hospitalier Universitaire de Clermont-Ferrand, Clermont-Ferrand, 63003 France

**Keywords:** Blood transfusion, Cesarean, Maternal morbidity, Postpartum hemorrhage, Uterine atony

## Abstract

**Background:**

Most estimates of postpartum hemorrhage (PPH) are calculated from studies that use administrative or medical birth databases, and only a few from prospective observational studies.

Our principal objective was to estimate the incidence of PPH according to their severity (mild or severe) in vaginal deliveries (>500 mL, ≥1000 mL) and cesareans (>1000 mL and ≥1500 mL). The secondary objectives were to describe the incidence of PPH according to maternity unit characteristics, causes, and types of PPH management.

**Methods:**

This prospective observational study took place in French maternity wards. Women who gave birth at a term ≥ 22 weeks were eligible for the study. 182 maternity units participated in a study with prospective data collection from 1 February, 2011, to 31 July, 2011. The main outcome measure was PPH incidence.

**Results:**

PPH incidence after vaginal delivery was 3.36 % [95 % CI: 3.25–3.47 %] and after cesareans 2.83 % [95 % CI: 2.63–3.04 %]. The incidence of severe PPH after vaginal delivery was 1.11 % [95 % CI: 1.05–1.18 %] and after cesareans 1.00 % [95 % CI: 0.88–1.13 %]. This incidence rate varied according to maternity unit characteristics. The principal cause of PPH for both modes of delivery was uterine atony (57.7 % for vaginal births and 66.3 % for cesareans). Vascular embolization was more frequent among women with cesareans (10.0 vs. 2.9 %), who also required transfusions more often (44.4 vs 12.7 %).

**Conclusions:**

The incidence of PPH was lower than the rate expected from the literature. Effective treatment of uterine atony and optimizing the identification of blood loss remain important priorities.

**Electronic supplementary material:**

The online version of this article (doi:10.1186/s12884-016-1008-7) contains supplementary material, which is available to authorized users.

## Background

Postpartum hemorrhage (PPH) remains a major cause of maternal deaths worldwide [1, 2]. In France, PPH was the leading cause of death, responsible for 18 % of maternal deaths in the decade from 1998 to 2007 [3]. Hemorrhage-related events are among the most preventable causes of maternal death.

Previous studies have suggested that severe maternal morbidity may be a better indicator of the quality of obstetric care, particularly in developed countries where maternal mortality is rare [4–9]. Obstetric hemorrhage is the main cause of severe maternal morbidity [6, 7, 10]. There is, however, no universally accepted definition of PPH [11, 12]. Accordingly, definitions using various levels of estimated blood loss [7, 13–19], various quantities of transfused blood [7, 16], specific decreases in postpartum hemoglobin [7, 20], or composite criteria [16, 20–22] have all been used in previous publications.

The prevalence of PPH (defined as ≥500 mL blood loss) and severe PPH (defined as ≥1 000 mL blood loss) are reported to be around 6.0 and 1.86 % of all deliveries, respectively, and vary widely throughout the world [23]. Most estimates are calculated from observational studies that use administrative or medical birth databases [14, 17–19, 24–29]; only a few come from prospective observational studies [15, 20, 30]. Of the two French population-based studies [20, 31], only one was prospective, conducted in 16 of the 33 maternity units of the Rhône-Alpes perinatal network in 2004–2005 [20]. The overall incidence of immediate PPH was 5.4 ± 0.3 %, but the diagnosis was clinical in 82.5 % of the severe cases and in 77.5 % of milder ones; the remainder were detected by postpartum laboratory tests [20]. A broader incidence study therefore appeared useful, one that would include several French regions, be supported by French perinatal networks, and for which all participating professionals would apply a standard definition of PPH.

The principal objective of this study was to estimate the incidence of PPH, as either relatively mild or severe, in vaginal (>500 mL and ≥1000 mL) and cesarean (>1000 mL and ≥1500 mL) deliveries. The secondary objectives were to describe the incidence of PPH according to characteristics of the maternity units, causes, and types of PPH management.

## Methods

### Materials

This prospective observational study was approved by a French institutional review board (Comité d’Ethique des Centres d’Investigation Clinique de l’Inter-région Rhônes-Alpes-Auvergne, Grenoble: CECIC): IRB 0917 on 9 November 2009]. Our study adhered to STROBE guidelines. All patients were informed about the study and that neither participation in it nor refusal to consent would affect their treatment, which would be the usual standard of care.

Eligible women had singleton or multiple pregnancies, regardless of parity, delivered stillborn or live born babies in a participating maternity unit by vaginal or cesarean delivery, at or after a gestation of 22 weeks (or, in the absence of a specific date for the beginning of the pregnancy, birth of a child ≥500 g).

### Data source

We contacted 43 French perinatal networks to request their support for this study of their maternity units. Twenty perinatal networks, covering 231 eligible maternity units, agreed to support this 6-month project (1 February 2011 to 31 July 2011). Finally, 182 maternity units participated, for a participation rate of 78.79 % maternity units (84 level I, 69 level II and 29 level III). In 10 perinatal networks (50 % of those participating), all of the network’s maternity units participated. Table 1 compares the characteristics of the French maternity units that participated and those that did not.

**Table 1:** Comparison of the characteristics of French maternity units that did and did not participate in the study

	Participants *n* = 181 %	Non-participants^a^ *n* = 377 %	*p* value
Total no. deliveries^b^			
< 250	7.7	11.4	0.12
250–749	58.0	49.9	
≥ 750	34.3	38.7	
Level of care^c^			
Level I	45.9	50.1	0.22
Level II	38.1	38.2	
Level III	16.0	11.7	
Type of facility			
University hospital	8.8	8.8	0.02
General hospital	64.1	52.5	
Private hospital	27.1	38.7	
Region^d^			
Province	82.9	82.8	0.97
Île de France	17.1	17.2	

^a^Maternity units that did not participate in the study among all French maternity units, according to 2010 healthcare facility statistics

^b^Delivery during the 6-month study period

^c^Level I: no neonatology department. Level II: presence of a department of neonatology and special care in the same building or immediate proximity to the site of delivery. Level III: neonatal intensive care present in the same building (in addition to neonatology units) or immediate proximity to the delivery room

^d^Ile de France includes Paris and its metropolitan area. *Province* is all other French regions

In each case of postpartum hemorrhage (defined as blood loss >500 mL for vaginal delivery and >1000 mL for cesareans in the 24 h after delivery), the medical and/or surgical treatment and maternal outcomes were recorded. Blood loss was to be estimated visually with the habitual method used in each unit and specified in each woman’s records. Professionals in each unit collected data prospectively for 6 months, entering it onto electronic case report forms via a secure website.

The principal endpoint was the incidence of PPH.

Quality control was performed in 30 % of the maternity units in each participating network, selected by random drawing, from September 2011 to November 2012. In each of the selected maternity wards, 10 % of the study files (again, randomly selected) were verified manually as were the birth registers, either by a supervisor of the perinatal network or by one of the two midwives coordinating the study nationwide. One maternity ward was excluded from the statistical analysis because it refused to participate in the quality control procedure. Finally 53 maternity units were audited (and 108 records verified).

### Statistical analysis

The χ2 test (or Fisher’s exact test when appropriate) was used to compare the qualitative variables and Student’s t test for the quantitative variables. Clinically relevant crude relative risks (RR) were calculated (cesareans compared with vaginal deliveries), with their 95 % confidence intervals (95 % CI). The analysis was performed with STATA software (version 13, Statacorp, College Station, TX, US). A *P* value < .05 was considered significant.

## Results

Table 2 describes the women’s medical characteristics. Women in the cesarean group were older, gave birth at an earlier gestational age, and had more multiple pregnancies (*P* < .001).

**Table 2:** Description of medical data of women who had a PPH

Women with PPH	Vaginal delivery and PPH *n* = 3488 % [mean ± SD]	Cesarean and PPH *n* = 719 % [mean ± SD]	*p* value
Delivery at term (weeks)	[39.7 ± 2.2]	[38.3 ± 3.1]	<.0001
Singletons	96.8	87.7	<.0001
Women’s age			
< 18 years	0.8	0.1	<.0001
18–35 years	83.5	70.7	
≥ 35 years	15.7	29.2	
Hemoglobin before delivery (mL)	*n* = 3335 [11.9 ± 1.1]	*n* = 699 [11.7 ± 1.2]	<.0001
Lowest postpartum hemoglobin (mL)	*n* = 3169 [9.0 ± 1.6]	*n* = 690 [8.2 ± 1.6]	<.0001
Total estimated blood loss (mL)	*n* = 3398 [895 ± 460]	*N* = 663 [1513 ± 816]	-
Estimated blood loss^a^			
Bag and/or aspiration and/or drains	*n* = 3465 90.4	*n* = 711 79.4	<.0001
Weighed	*n* = 3466 15.4	*n* = 710 20.7	.0005
Subjective measurement	*n* = 3464 21.5	*n* = 710 38.9	<.0001
Active management of third stage of labor^b^	*n* = 3469 79.8	*n* = 703 90.7	<.0001

^a^To participate in the study, blood loss had to be estimated visually but additional modes of estimation used in the maternity units were also considered. The estimate of blood loss could requires various combined methods of measurement, such as aspiration and the weighing of compresses during a cesarean

^b^Active management of the third stage of labor was defined as the use of uterotonic agents after childbirth. It was performed in 73.7 % of the cesareans with PPH immediately after birth and in 25.6 % of cases after delivery of the placenta. In vaginal deliveries, active management of the third stage of labor began at the emergence of the anterior shoulder in 91.5 % of cases, immediately after birth in 7.3, and in 0.9 % of cases after placental delivery

During the study period there were 129,110 deliveries in the participating maternity units (103,733 vaginal and 25,377 cesarean) with 4207 PPH reported, including 3488 after vaginal delivery, 714 PPH after cesareans, and 5 after a cesarean performed on the second twin.

The incidence of PPH (>500 mL) after vaginal delivery was 3.36 % [95 % CI: 3.25–3.47 %] and after cesareans (>1000 mL) 2.83 % [9 5% CI: 2.63–3.04 %]. The incidence of severe PPH after vaginal delivery (≥1000 mL) was 1.11 % [95 % CI: 1.05–1.18] and after cesareans (≥1500 mL) 1.0 % [95 % CI: 0.88–1.13 %].

For vaginal deliveries, the incidence of transfusions among the women with mild PPH (>500 mL and < 1000 mL) was 4.95 % [95 % CI: 4.08–5.93], and among those with severe PPH 28.45 % [95 % CI: 25.87–31.13]. For the cesareans, the incidence of transfusions with mild PPH (>1000 mL and < 1500 mL) was 33.16 % [95 % CI: 28.54–38.05] and among those with severe PPH 64.54 % [95 % CI: 58.28–70.46].

The incidence of PPH varied according to maternity unit characteristics (number of deliveries, type of hospital, and region) for cesareans (*P* < .003) and for vaginal deliveries (*P* < .001) (except for the number of deliveries) (Table 3).

**Table 3:** Incidence of PPH according to maternity unit characteristics

	Vaginal deliveries		Cesareans	
	*n* = 103,733 % [95 % CI]	*p* value	*n* = 25,377 % [95 % CI]	*p* value
No. deliveries^a^				
< 250	3.84 [3.07–4.73]	.11	1.31 [0.53–2.69]	<.001
250–749	3.23 [3.06–3.41]		2.20 [1.92–2.51]	
> 750	3.43 [3.29–3.58]		3.29 [3.01–3.59]	
Level^b^				
I	2.77 [2.58–2.96]	< .001	1.94 [1.63–2.29]	<.001
II	3.26 [3.09–3.44]		2.50 [2.21–2.83]	
III	4.05 [3.83–4.27]		4.05 [3.63–4.51]	
Region^c^				
Ile de France	2.78 [2.57–3.01]	< .001	2.27 [1.91–2.68]	.003
Province	3.52 [3.39–3.65]		3.01 [2.77–3.26]	

^a^Number of deliveries during the 6-month study period

^b^Level I: no neonatology department. Level II: presence of a department of neonatology and special care in the same building or immediate proximity to the site of delivery. Level III: neonatal intensive care present in the same building (in addition to neonatology units) or immediate proximity to the delivery room

^c^Ile de France includes Paris and its metropolitan area. Province: all other French regions

The principal cause of PPH for both modes of delivery was uterine atony (57.7 % for vaginal births and 66.3 % for cesareans) (Table 4). The second and third leading causes of PPH after vaginal delivery were, respectively, placental retention (38.7 %) and perineal tears and lacerations (24.9 %). For cesareans, the second leading cause was surgical complications (16.4 %) and the third abnormalities of placental insertion (12.7 %). Finally, in 8.5 % of the PPH after cesareans, the cause was not determined (vs. 1.6 % in vaginal deliveries). The risk of uterine atony was 1.15 times more frequent [95%CI: 1.08–1.22] and the risk of intraoperative complications 190 times more frequent [95 % CI: 60.84–598.46] for cesarean compared with vaginal deliveries (Table 4). When we limited the comparison of PPH for the 2 types of delivery by defining them by the same volume of blood loss (>1000 mL), the results pointed in the same direction (except for uterine atony, which no longer differed according to mode of delivery)[data not shown] (see Additional file 1: Table S1).

**Table 4:** Causes of PPH according to mode of delivery

Causes of PPH^a^	Vaginal delivery and PPH *n* = 3488 %^a^	Cesarean and PPH *n* = 719 %^a^	Crude RR^b^ [95 % CI]	*p* value
Uterine atony	57.7	66.3	1.15 [1.08–1.22]	<.0001
Placental retention	38.7	2.6	0.07 [0.04–0.11]	<.0001
Vaginal and/or perineal lacerations	24.9	1.7	-	-
Episiotomy	19.4	0.8	-	-
Anomaly of placental insertion	1.9	12.7	-	-
Uterine rupture^c^	0.4	1.7	4.15 [1.93–8.95]	-
Cervical lacerations	3.4	0.8	-	-
Vaginal thrombus	1.3	0.4^c^	-	-
Others				
intraoperative complications	0.1	16.4	190.81 [60.84–598.46]	<.0001
hemorrhagic normal 3rd stage^d^	0.3	0.1	-	-
amniotic fluid embolism	0.1	0.1	-	-
uterine inversion	0.1	0.0	-	-
coagulation disorders	0.2	2.6	-	-
not determined^e^	1.6	8.5	-	-

^a^One woman could have had several causes that explain her PPH

^b^cesareans vs. vaginal deliveries

^c^There were 3 trials of vaginal delivery (1 woman with an episiotomy and placental retention, 1 woman with no other anomaly, and 1 woman with a uterine rupture and a vaginal laceration)

^d^Hemorrhagic normal third stage: excessive blood loss during a normal separation of the placenta from the uterine wall

^e^The professionals were unable to select a principal cause for the PPH

The estimate of blood loss was more often subjective for cesarean than vaginal deliveries (38.9 vs. 21.5 %) (*P* < .0001); similarly use of active management of the third stage of labor (injection of uterotonic agents) was more frequent for cesareans (90.7 vs. 79.8 %) (*P* < .0001) (Table 2). Oxytocin was the uterotonic used after cesareans in 86.7 % of the cases, with carbetocin used in 13.3 %. Oxytocin was used in 98.4 % of the PPH after vaginal deliveries.

Women with PPH after vaginal delivery received the following pharmacological products: oxytocin in 89.8 % of cases (*n* = 3473), prostaglandins in 33.0 % (*n* = 3472), fibrinogen in 2.8 % (*n* = 3473), factor VIIa in 0.5 % (*n* = 3472), and tranexamic acid in 5.1 % (*n* = 3473). After cesareans, they received oxytocin in 76.6 % (*n* = 709), prostaglandins in 57.1 % (*n* = 711), fibrinogen in 10.8 % (*n* = 711), factor VIIa in 1.1 % (*n* = 711), and tranexamic acid in 14.3 % (*n* = 711).

The non-pharmacological procedures, that is, second-line treatments, are described in Table 5. Manual uterine examination was reported in 80 % of the PPH after vaginal deliveries. Intrauterine balloon treatment was still infrequent during the study period. Vessel embolization by interventional imaging was 3.4 times [95 % CI: 2.58–4.63] more frequent among women with cesareans than those with vaginal deliveries (10.0 vs. 2.9 %). At the same time, surgical procedures were less frequent after cesarean than vaginal deliveries (25.5 vs. 42.6 %) [RR = 0.6; 95 % CI: 0.52–0.68]. After excluding surgical procedures of the perineum, the RR for surgical procedures was 5.36 [95 % CI:4.22–6.82] after cesarean compared with vaginal deliveries (23.5 vs. 4.4 %). Women with cesareans required transfusions more often (44.4 vs. 12.7 %) [RR = 3.5; 95 % CI: 3.11–3.95] (Table 5).

**Table 5:** Non-pharmaceutical curative second-line procedures performed for PPH

Non-pharmaceutical procedures	Vaginal delivery and PPH *n* = 3488 %	Cesarean and PPH *n* = 719 %	Crude RR^f^ [95 % CI]	*p* value
Manual uterine examination	80.0	-	-	-
Intrauterine balloon	0.9	1.0	1.06 [0.47–2.39]	.9
Radiologic artery embolization	2.9	10.0	3.46 [2.58–4.63]	<.0001
Surgical procedures^a^	42.6	25.5	0.60 [0.52–0.68]	<.0001
B-Lynch suture	0.3	3.9	-	-
Ho Cho suture	0.1	3.5	-	-
Hypogastric arterial ligation	0.3	6.5	-	-
Other vessel ligation	0.5	10.3	-	-
Cervical suture	3.0^b^	0.7	-	-
Suture of a vaginal laceration	39.9	2.5	-	-
Hysterectomy	0.4	5.6	-	-
Repair of uterine wound closure	-	2.8	-	-
Evacuation of hematoma of the abdominal wall	0	0.7	-	-
Other surgery	0.3^c^	6.1^d^	-	-
Transfusion of packed red blood cells	12.7	44.4	3.5 [3.11–3.95]	<.0001
Maternal death	0.03^e^	0	-	-

^a^Regardless of the type of surgical procedure

^b^Among women with a cervical laceration, we note 3 emergency hysterectomies

^c^Corresponds to 8 uterine ruptures sutured without a hysterectomy and one traction rotation of the cervix with forceps

^d^Corresponds to 3 uterine ruptures sutured without a hysterectomy and 6 sutured bladder lacerations ; 2 laparotomies with ablation of clots without active bleeding ; 1 laparatomy for a retroperitoneal hematoma, and a ruptured hepatic adenoma ; 2 laparotomies with reoperation of the uterus and evacuation of intrauterine clots

^e^One maternal death

^f^Cesareans vs. vaginal deliveries

Again, if we limit the comparison of the two types of delivery by defining PPH by the same volume of blood loss (>1000 mL), the results again pointed in the same direction, with arterial embolization still more frequent but no longer statistically significantly (10.0 % vs. 8.1 %; RR = 1.24 [95 % CI: 0.89–1.71] and transfusion of packed red blood cells [RR = 1.27; 95 % CI:1.12–1.44] more frequent for cesarean deliveries [data not shown] (see Additional file 2: Table S2).

## Discussion

### Study strengths and limitations

This study covered 129,110 of the 786,559 deliveries recorded in France in 2011 and accounted for 32.82 % of all deliveries during the 6 months of the study [32]. However, private hospitals participated at a lower rate than the other types of hospitals (Table 1). Among the 20 perinatal networks that agreed to support the study, the participation rate was satisfactory (78.79 %). This participation was total (100 % of their maternity units) for 50 % of the networks. The definition of PPH was standardized and the data collection prospective, in a daily clinical setting. Substantial quality control to verify data input quality was performed in 30 % of the maternity units.

The choice to use different definitions of PPH according to mode of delivery can be debated. Based on a discussion between obstetricians, it was intended to facilitate the participation of the obstetrics professionals in the maternity units. A large number of obstetricians considered that a threshold of 500 mL for cesareans was inappropriate, both because it would considerably increase the number of PPH among cesareans and because the amniotic fluid aspired at the moment of incision makes this threshold unreliable. Moreover, although the French guidelines of December 2014 clearly define PPH as blood loss ≥500 mL, regardless of mode of delivery, the definition was less clear in the 2004 guidelines, in effect during the study period [33, 34]. We further note that the American College of Obstetricians and Gynecologists uses the same definition we did: > 500 mL for vaginal deliveries and > 1000 mL for cesareans [35].

Another criticism is that the determination of the volume of blood loss was not more objective. Visual estimates of blood loss are known to be inaccurate and associated with an underestimation of blood loss [11, 36]. However, it is the first-line method available to physicians in the immediacy of decision making and it is widely used in French maternity wards. In a large European trial, the use of a collection bag compared with visual estimates of blood loss after vaginal delivery was not found to reduce the rate of severe PPH [37].

### Interpretation

The incidence of PPH (>500 mL) after vaginal delivery was 3.36 % [95 % CI: 3.25–3.47 %], slightly lower than the percentage reported in several studies [14, 19, 23]. However, the studies that measured blood loss objectively, as opposed to subjectively, have showed higher prevalence rates [23]. Elsewhere, there is a wide variation in PPH rates throughout the world [23]. The incidence of severe PPH (≥1000 mL) after vaginal delivery was 1.11 % [95 % CI: 1.05–1.18], lower than reported by some authors [17, 38] and similar to the results in another French study [20]. Here again, this prevalence varies widely internationally [23, 30]. The incidence of PPH (>1000 mL) after cesareans was 2.83 % [95 % CI: 2.63–3.04 %], similar to other reported rates [37, 39], and the incidence of severe PPH (≥1500 mL) after cesareans was 1.0 % [95 % CI: 0.88–1.13 %]. It is not always easy to compare the published studies because the definition of severe PPH varies from study to study and is not often defined simply as a volume of blood loss [20, 21, 25]. Finally, despite definitions of PPH that vary by mode of delivery, authors generally calculate an overall incidence of PPH without distinguishing the mode of delivery [12, 18, 29, 40–42]. The incidence of PPH here varied according to maternity unit level, contrary to the results in another French study [20]. The incidence of PPH varied in our study according to number of deliveries only for the cesareans; this is unsurprising because patients at risk of PPH are most often referred to Level III units, which are also the largest. Nonetheless, a US study found a higher rate of PPH in the lowest volume rural hospitals than in non-rural hospitals, for both cesareans and spontaneous vaginal deliveries [43].

Blood loss volume was measured subjectively in 21.5 % of the PPH after vaginal delivery, compared with 38.9 % of those after cesareans. This may explain in part the more frequent use in France of second-line treatments, which underlines a possible delay in diagnosis [14]. It should be noted that in France, most maternity units do not routinely perform blood counts, except in PPH identified by the volume of blood loss or by clinical signs of hypovolemia or anemia. Finally, active management of the third stage of labor was performed for only 79.8 % of the PPH after vaginal delivery and 90.7 % after cesareans, although the 2004 French guidelines recommended this management routinely for all deliveries [33]: active management of the third stage of labor, including the prophylactic application of uterotonics, is considered a key point in the prevention of PPH [44]. Oxytocin is the agent used most frequently, even after cesareans. The percentage of active management of the third stage of labor has increased in France since 2006 [45]. It is probable that the national rate in 2015 is still better, after the 2014 publication of the updated French guidelines. Our results are consistent with those from a database collecting information regularly from volunteer French maternity units [46].

Unsurprisingly, uterine atony was the most common cause of PPH both for vaginal and cesarean deliveries (Table 4), but our results highlight the frequency of multiple causes in the same patient. The third leading cause of PPH after vaginal delivery was perineal tears or lacerations, and the fourth leading cause episiotomies. Episiotomies remain relatively common in France, with an overall rate around 28.5 % [46] and a rate in nulliparas around 45 % [46, 47]. The second leading cause of PPH during cesareans in our study was related to the surgical incision (16.4 %). To our knowledge, this is the first study to detail the causes of PPH associated with surgical procedures among the cesareans with PPH. The extension of the uterine incision at cesarean delivery is well known to obstetricians [21, 48, 49], but other causes of PPH after a cesarean can include uterine bleeding due to incision into the muscle, active bleeding persisting after suture of the incision, and hematomas, either subperitoneal or of the broad ligament or the uterine wall, or due to an incision either transplacental or through a highly vascularized lower segment. The rate of relaparotomy after cesarean delivery is thought to be around 1.5–2 % [49–51]. Levin et al. found that 89.3 % of the indications for relaparotomy after cesareans were associated with hemorrhagic complications (28 relaparotomies for 17,482 cesareans) [50]. The surgical incision during cesareans is therefore associated with immediate or secondary hemorrhagic complications, to which we must add non-hemorrhagic intraoperative or postoperative complications [48, 52, 53]. These risks of immediate (not to mention longer-term) complications must encourage obstetricians to limit the number of cesareans and therefore to avoid choosing to perform first cesareans, especially among nulliparous women.

The pharmacologic treatments used in this study show notable use of tranexamic acid, fibrinogen, and recombinant activated factor VII; they were used more often after cesareans and signal that these deliveries entailed more serious PPH.

Among the non-pharmacological curative second-line procedures, we see that the intrauterine balloon tamponade was still little used in France at that time, unlike in UK maternity units [54]. Use of balloon tamponade is relatively recent in France, and the first small French series was not published until 2012 [55]. This technique has since been spreading among French maternity units and is now proposed after failure of prostaglandin treatment by the 2014 French guidelines [34]. We also note that embolization is fairly frequent in France, particularly after cesareans, somewhat surprisingly. That is, one would expect more revision surgery after cesareans, but that may be explained by the delayed character of PPH in the recovery room or during postpartum monitoring, and by easy access to vascular embolization platforms in larger maternity units, outside the Paris region. Emergency hysterectomy was, as in the literature, more frequent after cesareans (5.6 %) [28, 56–59]. Second-line treatment of PPH remains challenging, since we lack sufficient scientific evidence from randomized controlled trials for choosing the specific treatment.

After vaginal deliveries, 12.7 % of the women with PPH had transfusions (vs. 44.4 % for the cesareans with PPH). The transfusion rate after vaginal deliveries reported here is higher than that observed in other studies [14, 60]. The excess risk of transfusion after cesareans also varies according to parity, number of previous cesareans, and type of cesarean (elective vs. emergency), but these factors were not collected in this study [61–63].

## Conclusion

The incidence of PPH after vaginal delivery was 3.36 % and after a cesarean 2.83 %. The incidence of severe PPH after vaginal delivery was 1.11 % and after a cesarean 1.00 %. This incidence rate varied according to maternity unit characteristics. The principal cause of PPH for both modes of delivery was uterine atony, which is therefore the obstetric complication for which improvement in prevention, identification, and management remain important priorities in maternity units.

It is essential to have harmonized international definitions of PPH after vaginal and cesarean deliveries, whether it is volume or method of collection of blood loss, in order to facilitate the comparison of the incidence and prevalence rates of PPH in both developed and low-income countries. Moreover, studies should seek to assess the methods to optimize immediate diagnosis of PPH, such as improvements in the visual estimates of blood loss (simulations of clinical scenarios, posters with photographs of blood losses accompanied by a calibrator to help determine the blood volume) or weighing the lost blood.

## Abbreviations

CECIC, Comité d’Ethique des Centres d’Investigation Clinique; HERA Group, [postpartum HEmoRrhAge] group; IRB, Institutional review board; PPH, Postpartum hemorrhage

## Additional files

Additional file 1: Table S1. Description of data: Causes of PPH >1000 mL according to mode of delivery. (DOCX 18 kb) Additional file 2: Table S2. Description of data: Non-pharmaceutical curative second-line procedures performed for PPH > 1000 mL. (DOCX 19 kb)
