# Supplementary material for: Incidence of immediate postpartum hemorrhages in French maternity units: a prospective observational study (HERA study)
Source: BMC Pregnancy Childbirth. 2016 Aug 24;16(1):242. doi: 10.1186/s12884-016-1008-7 (PMC4995746; doi:10.1186/s12884-016-1008-7)
Supplement: Additional file 1: Table S1. — Description of data: Causes of PPH >1000 mL according to mode of delivery. (DOCX 18 kb) [file 12884_2016_1008_MOESM1_ESM.docx]

**Supplemental data 1:
Table S1 Causes of PPH >1000 mL according to mode of delivery**

| **Causes of PPH** | **Vaginal delivery and PPH  n= 765 %^a^** | **Cesarean and PPH n=719 %^a^** | **Crude RR^b^ [95%CI]** | **p value** |
| --- | --- | --- | --- | --- |
| **Uterine atony** | 66.3 | 66.3 | 1.00 [0.93-1.08] | 0.99 |
| **Placental retention** | 41.2 | 2.6 | 0.06 [0.04-0.09] | <.0001 |
| **Vaginal and/or perineal lacerations** | 23.1 | 1.7 | - | - |
| **Episiotomy** | 17.5 | 0.8 | - | - |
| **Anomaly of placental insertion** | 2.8 | 12.7 | - | - |
| **Uterine rupture** | 1.1 | 1.7 | 1.6 [0.66-3.88] | 0.30 |
| **Cervical lacerations** | 5.2 | 0.8 | - | - |
| **Vaginal thrombus** | 1.8 | 0.4^c^ | - | - |
| **Others** intraoperative complications hemorrhagic normal 3^rd^ stage ^c^ amniotic fluid embolism uterine inversion  coagulation disorders  not determined^d^ | 0.3 0.3 0.3  0.3 0.7 0.5 | 16.4 0.1 0.1  0.0 2.6 8.5 | 62.8 [15.6-253.0] - - -  - - | <.0001 - -  - - - |

^a^One woman could have had several causes that explain her PPH.
^b^Cesareans vs. vaginal deliveries. ^c^Hemorrhagic normal third stage: excessive blood loss during a normal separation of the placenta from the uterine wall.

^d^The professionals were unable to select a principal cause for the PPH.
