# Supplementary material for: Incidence of immediate postpartum hemorrhages in French maternity units: a prospective observational study (HERA study)
Source: BMC Pregnancy Childbirth. 2016 Aug 24;16(1):242. doi: 10.1186/s12884-016-1008-7 (PMC4995746; doi:10.1186/s12884-016-1008-7)
Supplement: Additional file 2: Table S2. — Description of data: Non-pharmaceutical curative second-line procedures performed for PPH > 1000 mL. (DOCX 19 kb) [file 12884_2016_1008_MOESM2_ESM.docx]

**Supplemental data 2
Table S2 Non-pharmaceutical curative second-line procedures performed for PPH > 1000 mL**

| **Non-pharmaceutical procedures** | **Vaginal delivery and PPH n=765 %** | **Cesarean and PPH n=719 %** | **Crude RR^b^ [95%CI]** | **p value** |
| --- | --- | --- | --- | --- |
| **Manual uterine examination** | 91.5 | - | - | - |
| **Intrauterine balloon** | 2.6 | 1.0 | 0.37 [0.16-0.88] | .02 |
| **Radiologic artery embolization** | 8.1 | 10.0 | 1.24 [0.89-1.71] | .21 |
| **Surgical procedures^a^**  B-Lynch suture  Ho Cho suture  Hypogastric arterial ligation  Other vessel ligation  Cervical suture  Suture of a vaginal laceration  Hysterectomy  Repair of uterine wound closure  Evacuation of hematoma of the abdominal wall  Other surgery | 42.1 1.2  0.4 1.1 1.6 5.1 36.9 1.7 - 0  0.5 | 25.5 3.9  3.5  6.5 10.3 0.7 2.5 5.6  2.8 0.7  6.1 | 0.60 [0.52-0.70]^c^ - - - - - - - - -  - | <.0001  - -  -  -  -  -  -  -  -  - |
| **Transfusion of packed red blood cells** | 34.9 | 44.4 | 1.27 [1.12-1.44] | .0002 |
| **Maternal death** | 0.1 | 0 | - | - |

^a^Regardless of the type of surgical procedure.

^b^Cesareans vs. vaginal deliveries

^c^After exclusion of perineal surgical procedures, the RR of surgical procedures was 2.84 [95%CI: 4.05-3.94] after cesareans compared with vaginal deliveries (23.5% vs.8.3%).
